# Ethanol extract of *Ligustrum lucidum Ait.* leaves suppressed hepatocellular carcinoma in vitro and in vivo

**DOI:** 10.1186/s12935-019-0960-5

**Published:** 2019-09-26

**Authors:** Guoyan Tian, Jin Chen, Yan Luo, Jin Yang, Tao Gao, Junping Shi

**Affiliations:** 1 Department of Oncology and Hematology, The Affiliated Hospital of Hangzhou Normal University, Hangzhou, Zhejiang Province China; 2 The Fourth Clinical Medical College, Zhejiang Chinese Medicine University, Hangzhou, Zhejiang Province China; 3 Institute of Translational Medicine, The Affiliated Hospital of Hangzhou Normal University, Hangzhou, Zhejiang Province China; 4 TCM Gynecology, Hangzhou Hospital of Traditional Chinese Medicine, Hangzhou, Zhejiang Province China; 5 Department of Liver Diseases, The Affiliated Hospital of Hangzhou Normal University, Hangzhou, Zhejiang Province China

**Keywords:** Ethanol extract of *Ligustrum lucidum Ait*. leaves (EEL), Hepatocellular carcinoma (HCC), DNA methylation

## Abstract

**Background:**

The present study investigated the pharmacological activity and mechanism of ethanol extract of *Ligustrum lucidum Ait.* leaves (EEL) on HCC.

**Methods:**

Cell viability was determined using cell counting kit-8 (CCK-8) assay. The effects of EEL on cellular biological activities were analyzed by flow cytometry (FCM), cell wound scratch assay and transwell assay. The expression levels of related mRNA and protein were determined by performing quantitative real-time PCR (qRT-PCR), Western blotting assay and immunocytochemistry. Methylation-specific PCR (MSP) was carried out to investigate the possible mechanism underlying the DNA methylation of PTEN.

**Results:**

EEL showed cytotoxicity to both Bel-7402 and Huh-7 cell lines. We also found that EEL enhanced the apoptosis of Bel-7402 and Huh-7 cells by regulating the expressions of Bcl-2 associated X (Bax), B cell lymphoma 2 (Bcl-2) and Cytochrome-C and the activity of caspase-3 and therefore promoted cell cycle arrest. Moreover, EEL also suppressed cell migration and invasion. EEL increased the expression of tissue inhibitor of metalloproteinases 2 (TIMP2) but decreased the expressions of matrix metalloproteinase2 (MMP2) and MMP9. Furthermore, EEL inhibited the phosphorylation of PI3K/Akt pathway. MSP results showed that EEL promoted the demethylation of PTEN, suggesting that the inactivation of PI3K/Akt may be related to DNA de-methylation of PTEN. In addition, EEL inhibited the tumor growth of HCC in vivo.

**Conclusions:**

EEL exerted anti-tumor effect on HCC in vitro and in vivo. EEL mediated by the inhibition of PI3K/Akt may be closely related to DNA de-methylation of PTEN. Thus, EEL could be used as a potential anti-cancer therapeutic agent of HCC.

## Background

Hepatocellular carcinoma (HCC) has the second highest mortality rate worldwide [1]. Chemotherapy, radiotherapy and surgery, which are main strategies for treating HCC, are effective in treating HCC at the beginning of the therapy, however, recurrence of HCC still remains a challenge for the treatment of HCC. Moreover, as first-line drugs for HCC patients, platinum-based drugs have anti-tumour effects but would still cause gastrointestinal reaction, anaphylaxis, myelosuppression, kidney damage and neurovirulence [2, 3]. Thus, discovering novel therapeutic targets and therapeutic strategies for HCC is still fascinating and significant.

Molecular target therapy in cancer treatment has attracted much attention and it is also necessary to further understand the molecular mechanism of HCC progression. Previous researches showed that the PI3K/PTEN/Akt pathway was closely involved in malignant tumors such as hepatocellular carcinoma, hematological neoplasms and lymphoma [4–7]. PI3K phosphorylates PIP2, and generates PIP3 which is then used by PDK1/PDPK1 (3-phosphoinositide-dependent protein kinase 1) to phosphorylate AKT. AKT is a protein kinase named also protein kinase B or PKB [8, 9]. As the first discovered cancer suppressor gene with bispecific phosphatase activity, PTEN has been reported to be able to inhibit the activation of Phosphoinositide 3-kinase (PI3K) and therefore affects the phosphorylation level of its downstream target Akt [10].

Ethanol extract of *Ligustrum lucidum Ait.* leaves (EEL) is usually used as restoratives for liver and kidney in traditional Chinese medicine prescription. The main elements of EEL might be total phenylpropanoid glycosides, secoiridoids, which might have protective effects on anti-osteoporosis, and bone marrow suppression [11–13], EEL has multiple pharmacological activities, for example, anti-bacterial and anti-fungal activities and inhibiting hypoxia-induced retinal angiogenesis [14, 15]. Recently, a study showed that *Ligustrum lucidum Ait.* fruit extract was able to induce cell apoptosis and increase cell senescence in human HCC cell line by up-regulating the expression of p21. The findings by Hu et al. [16] also developed our interests to investigate therapeutic effect of EEL on HCC and its mechanism. To the best of our knowledge, the connection of EEL and PTEN/PI3K/Akt signaling pathway has not been completely studied. Therefore, in this study, we aimed to verify the possible pharmacological activity of EEL on HCC and to explore the underlying mechanism. Our study provided a novel therapeutic strategy to protect against HCC.

## Materials and methods

### Cells and reagents

Bel-7402 and Huh-7 cell lines were purchased from BeNa Culture Collection. Cells were cultured in RPMI-1640 medium with 10% FBS and 1% pen-strep, at 37 °C in a humidified incubator with a 5% CO_2_ atmosphere. All products used in cell culturing were purchased from Gibco (Carlsbad, CA,USA). EEL power was obtained from CR Sanjiu (Shenzhen, Guangdong, China), and dissolved in RPMI-1640 medium and adjusted to a concentration of 400 mg/ml, SC79 (Beyotime, China) is the Akt activator which could enhance Akt phosphorylation and its kinase activity.

### Cell counting kit-8 (CCK-8) assay

Cell viability was determined by CCK-8 (BeyotimeBio, Shanghai, China) assay. Cells in the logarithmic phase were seeded into 96-well plates (approximately 5 × 10^4^ cells/well) and maintained for 12 h in the incubator (37 °C, 5% CO_2_). Next, EELs at different concentrations (5, 10, 25, 50, 75 and 100 mg/ml) were incubated with the cells in the incubator (37 °C, 5% CO_2_) for 48 h. Additionally, 75 mg/ml EEL (EEL3 group), or 400 mg/ml SC79 (SC group), or 75 mg/ml EEL and 400 mg/ml SC79 (EEL3 + SC group) were incubated with the cells in the incubator (37 °C, 5% CO_2_) for 48 h. Enzyme labeling instrument was used to read the absorbance at 450 nm. Cell viability was determined by the proportion of survived cell in comparison with the control. IC50 values were calculated by the Bliss method (n = 6). All experiments were performed independently in triplicate.

### Flow cytometry (FCM)

Following the instructions, cell cycle and apoptosis detection kit (Beyotime, China) were used for cell cycle and apoptosis measurement. For cell cycle detection, cells were then re-suspended in PI staining solution for 30 min at 37 °C. For apoptosis detection, cells were stained with 5 μM Annexin V-FITC at 4 °C for 15 min, fixed in 70% ethanol overnight at 4 °C and then washed by PBS. The cells were then re-suspended in PI staining solution for 30 min at 37 °C. FACSCalibur flow meter with CellQuest software 3.2 (BD Biosciences) was used for data analysis.

### Real-time quantitive PCR (qRT-PCR)

The expression levels of Bcl-2 Assaciated X (Bax), B cell lymphoma 2 (Bcl-2), Cytochrome-C, Ki67, Cyclin D1, p21, Tissue inhibitor of metalloproteinases 2 (TIMP2), matrix metalloproteinase 2 (MMP2) and MMP9 mRNA were detected by performing qRT-PCR. The cells were seeded into 6-well plates at a density of 2 × 10^6^ cells/well. Total RNA was extracted using Trizol (Thermo Fisher Scientific Inc, New York, USA) following the manufacture’s instruction. cDNA was synthesized by M-MLV reverse transcriptase (Promega, USA). Glyceraldehyde-3-phosphate dehydrogenase (GAPDH) was used as the internal control to monitor the efficiency of qRT-PCR. All primers used in this study were synthesized by Shanghai Sangon Biotech Co. Ltd. (Shanghai, China). Specific primer sequences for each gene were listed as follows: 5′ GCCAGCAAACTGGTGCTCAA 3′ and 5′ CCAACCACCCTGGTCTTGGA 3′ for Bax; 5′ CACTGGCCAGGGTCAGAGTT 3′ and 5′ TGGCCATAGACCCTGTCAGC 3′ for Bcl-2; 5′ TGTCCAGAAATGTTCCCAGTGC 3′ and 5′ CCTTTGTTCTTATTGGCATCTGTG 3′ for Cytochrome-C; 5′ GAACAAACAGATCATCCGCAA 3′ and 5′ CCCTTCTGGTATCAAAATGC 3′ for Cyclin D1; 5′ CTGTGCGCAGATTCACGGAGA 3′ and 5′ ACAAAGTCGAAGTTCCACCGC 3′ forp21; 5′ TTCAAAGGGCCTGAGAAGGA 3′ and 5′ TCAGGCTCTTCTTCTGGGTG 3′ for TIMP2; 5′ GATACCCCTTTGACGGTAAGGA 3′ and 5′ CCTTCTCCCAAGGTCCATAGC 3′ for MMP2; 5′ TTCAGGGAGACGCCCATTTC 3′ and 5′ AAACCGAGTTGGAACCACGA 3′ for MMP9; 5′ GCCATCACAGCAACACAGAA 3′ and 5′ GCCATACCAGTAAGCTTGCC 3′ for GAPDH.

### Western blotting assay

Cultured cells were lysed on ice in RIPA lysis buffer (Thermo Scientific, 89900). The cells were broken into pieces by using an ultrasonic cell disruptor. The protein level of the cells was detected using a protein assay reagent (Bio-Rad Laboratory, Hercules, CA, USA) following the explanatory memorandum. Proteins at Equal quantity (50 μg) from each sample were separated by 8% sodium dodecyl sulfatepolyacrylamide gel electrophoresis (SDS-PAGE). Next, the proteins were transferred onto nitrocellulose membranes (Millipore, Billerica, MA, USA), and the nonspecific sites were blocked by immersing the membranes into 5% low skimmed milk at room temperature for 2 h. The membranes were incubated with primary antibodies (anti-Bax, Dilution, 1:1000, Abcam, ab32503; anti-Bcl-2,Dilution, 1:500, Abcam, ab692;anti-Cytochrome-C, Dilution, 1:1000, Abcam, ab13575; anti-Ki67, Dilution, 1:800, Abcam, ab16667; anti-CyclinD1, Dilution, 1:10000, Abcam, ab134175; anti-p21, Dilution, 1:1000, Abcam, ab109199; anti-TIMP2, Dilution, 1:1000, Abcam, ab1828; anti-MMP2, Dilution, 1:800, Abcam, ab37150; anti-MMP9, Dilution, 1:800, Abcam, ab38898; anti-p-PI3K, Dilution, 1:800, Abcam, ab182651; anti-PI3K, Dilution, 1:1000, Abcam, ab189403; anti-p-Akt, Dilution, 1:500, Abcam, ab38449; anti-Akt, Dilution, 1:6000, Abcam,ab81283) against IL-11 (1:600, SantaCruz, CA, USA) or IL-11Rα (1: 500, SantaCruz, CA, USA) overnight at 4 °C. The membranes were cultured with HRP-coupled secondary antibodies (ab6789, 1:2000, Abcam) at room temperature for 1 h. The blots were developed using enhanced chemiluminescent reagents (Millipore, Bedford, MA, USA). The gray value for the blots was analyzed by Quantity One software version 4.6.9 (Bio-Rad Laboratories).

### Cell wound scratch assay

Horizontal line was drawn at the back of 6-well plates using a marker pen, while 200 μl tips were used to draw horizontal lines at the bottom of plates. The distance was determined by Image-Pro Plus 6.0 software (Media Cybernetics Inc., Rockville, MD). Calculation formula is: the inhibition rate of cell migration (%) = (1 − migration distance of experimental groups/migration distance of the control group) × 100%.

### Transwell assay

Cell invasion were determined by transwell assay with matrigel. The cells were suspended in RPMI-1640 medium without FBS and adjusted to a concentration at 2 × 105 cells/ml. Cells suspensions (100 μl) were added onto the polycarbonate membrane of upper chamber with Matrigel (BD Bioscience, San Diego, CA). The bottom chamber was filled with complete RPMI-1640 medium (500 μl). The cells were incubated at 37 °C for 24 h. The cells on the bottom of the coated transwell were washed twice, fixed with 4% paraformaldehyde (Cat#P6148) for 30 min and stained with 0.1% Crystal Violet (No. C3886-100G0; Sigma-Aldrich, St. Louis, MO) at room temperature for 15 min. The invaded cells were analyzed from five randomly selected fields under a microscope at a magnification of ×100.

### Methylation-specific PCR (MSP)

The modified DNA samples were amplified by MSP assay with specific primers for to distinguish methylated (M) DNA from unmethylated (U) DNA. Water without DNA template was treated as the negative control. In all sodium bisulfite conversion reactions, the universal human methylated DNA standards from Zymo Research (ZYMO Research, Freiburg, Germany) was regarded as positive methylated controls, whereas DNA from normal lymphocytes was treated as negative control for methylated alleles of PTEN. The PCR products were analyzed by electrophoresis in 1.5% agarose gel and then stained with GelRed (Biotium, Belgium). Finally, PCR products were visualized under ultraviolet illumination.

### Animal study

The animal study was approved by Animal Ethics Committee of The Affiliated Hospital of Hangzhou Normal University. BALB/c nude mice (approximately 3–4 weeks old, weighted 12–15 g, female) in this study were purchased from Medical Laboratory Animal Center of Guangdong Province (China). The animals were fed according to IACUC, after liver tumor cell lines were subcutaneously injected into the flanks of the mice. After 2 weeks of acclimation, EEL (1.5 g/kg, 3 g/kg, 5 g/kg) were suspended in PBS (1 × 10^5^/50 μl) and then subcutaneously injected into the flanks of the mice daily. The animals were euthanized when they became moribund at 4 weeks. The tumor tissues were collected and the diameter of each tumor was measured.

### Statistical analysis

All results were shown as mean ± SD for at least three independent experiments. Results of all assays were analyzed by one-way analysis of variance (ANOVA) following Turkey’s test. Statistical significance was defined as *P *< 0.05.

## Results

### EEL decreased the cell viability of Bel-7402 and Huh-7

CCK-8 assay was carried out to evaluate the cytotoxicity of EEL on HCC cells, the results showed that EEL produced limited cytotoxicity to HCC cells at the concentration of 5 mg/ml. In comparison with control group, cell viability began to decrease at the concentration of 10 mg/ml after 48 h but significantly decreased by EEL at 25 mg/ml after 24 h (Additional file 1: Figure S1). Thus, EEL at the concentrations of 25 (EEL1 group), 50 (EEL2 group) and 75 (EEL3 group) mg/ml were used to treat cells for 24 h for the subsequent experiments. It might be a limitation not showing data at 0 h, as it was of no change and difference, and commonly not showing.

### EEL promoted apoptosis and cell cycle arrest of Bel-7402 and Huh-7

**Fig. 1:**
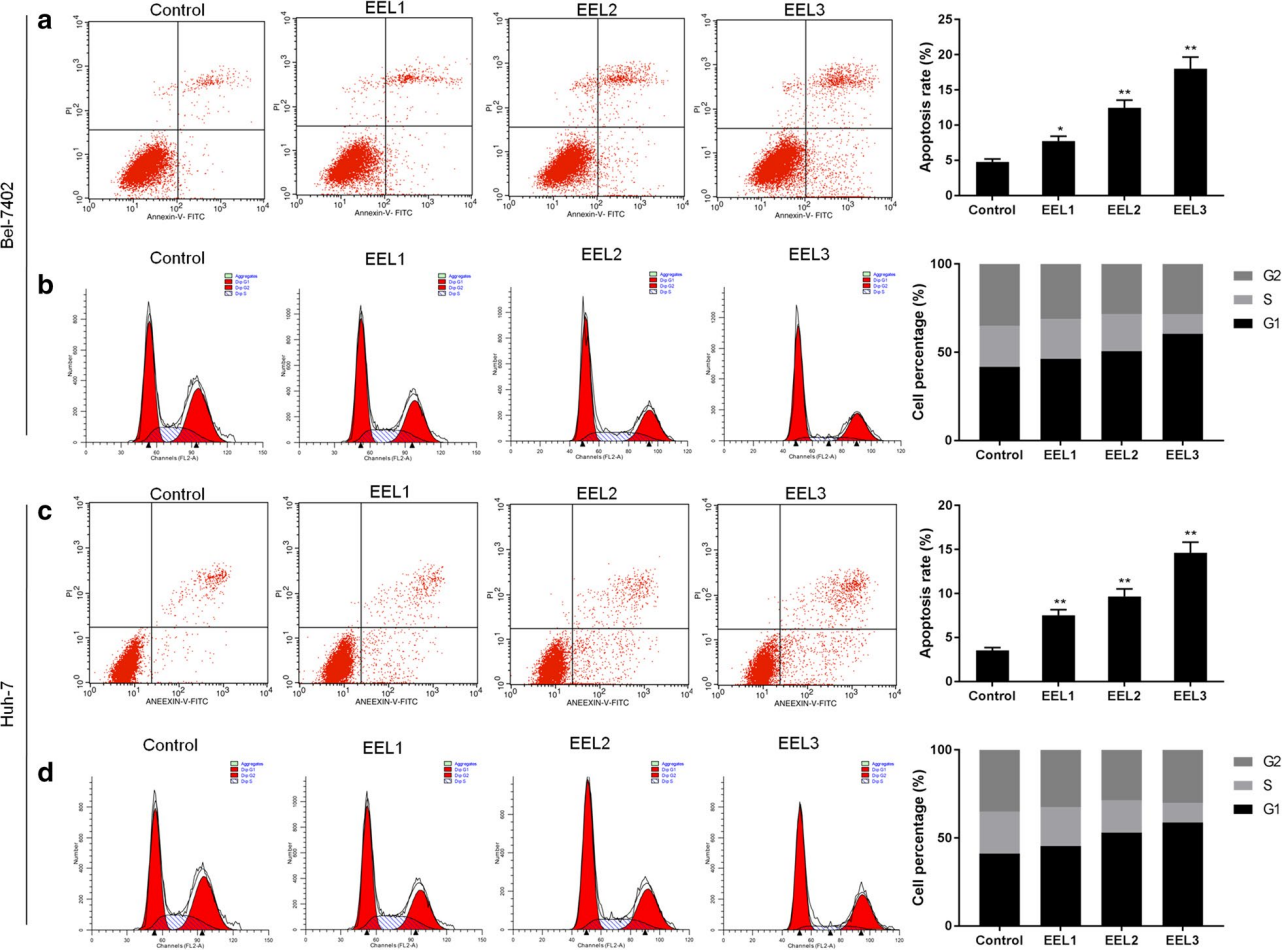
Effect of EEL treatment on cell apoptosis and cell cycle distribution. Bel-7402 and Huh-7 cells in control, EEL1, EEL2 and EEL3 groups were respectively treated with 0, 25, 50 and 75 mg/ml EEL for 24 h. **a**, **b** Cell apoptosis and cell cycle distribution in Bel-7402 were detected using FCM. **c**, **d** Cell apoptosis and cell cycle distribution in Huh-7 were detected using FCM. Data were shown as mean ± S.D. for three independent experiments. ^*^P < 0.05, ^**^P < 0.01 versus control

We further investigated whether EEL affected the apoptosis of Bel-7402 and Huh-7 cells. Data from FCM demonstrated that the proportions of apoptotic cells of Bel-7402 cell line in control groups were 3.62% and 4.87%, however, the proportion of apoptotic cells increased significantly in a dose-dependent manner in EEL-treated groups (Fig. 1a, c). Moreover, data from FCM showed that nearly 40% cells were in the G1 phases in the control group. The EEL treatment noticeably increased the proportion of cells in G1 phase as the concentration of EEL increased, suggesting that EEL could promote G1 phase cell cycle arrest of Bel-7402 and Huh-7 cells (Fig. 1b, d).

### EEL modulated the expression of apoptosis- and cell cycle arrest- associated proteins

**Fig. 2:**
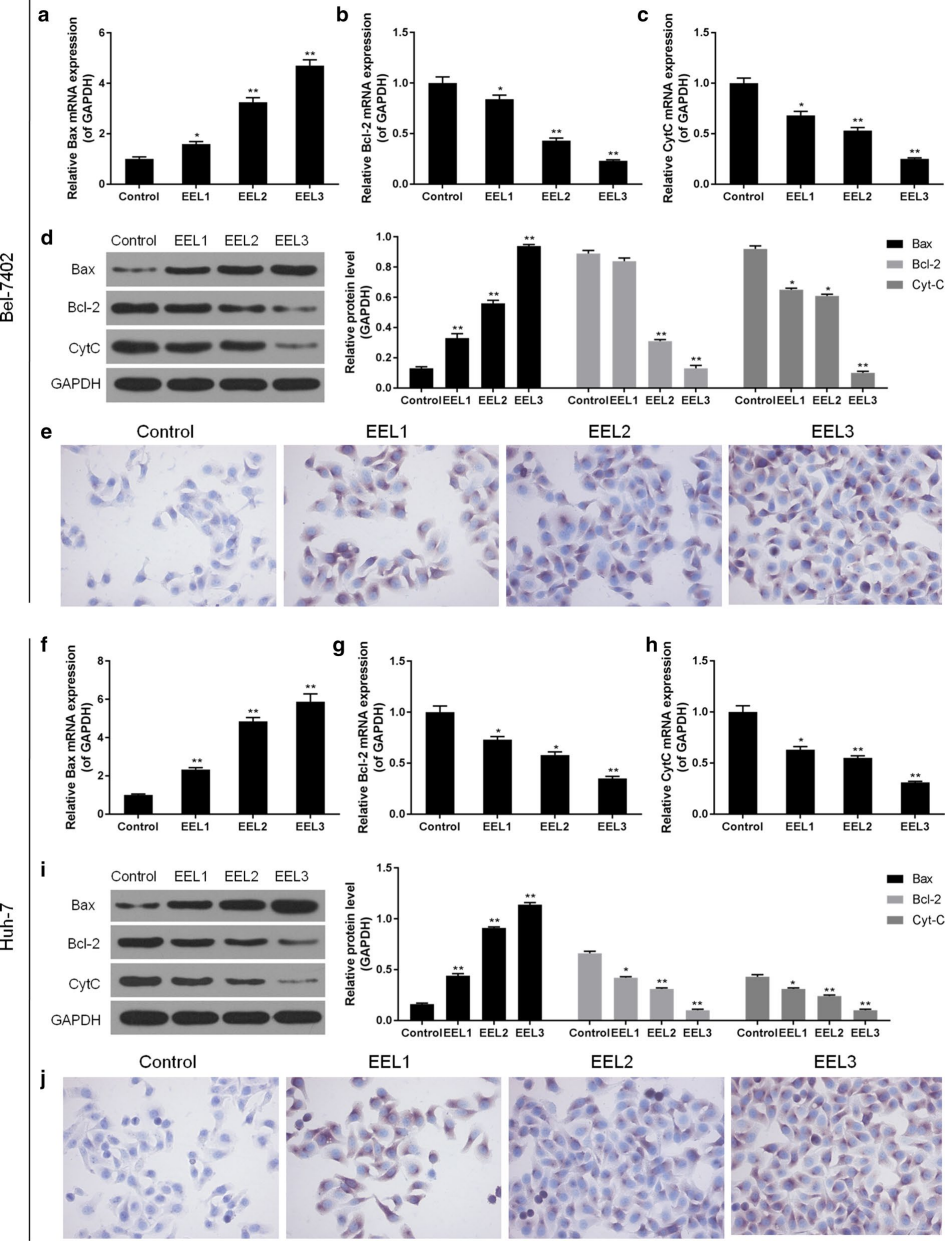
Effect of EEL treatment on the expression levels of apoptosis-related genes. Bel-7402 and Huh-7 cells in control, EEL1, EEL2 and EEL3 groups were respectively treated with 0, 25, 50 and 75 mg/ml EEL for 24 h. Data in **a**–**e** were generated with Bel-7402 cells, while data in F-J were produced with Huh-7 cells. **a**–**c** mRNA levels of Bax, Bcl-2 and Cyt-C in Bel-7402 were determined using RT-qPCR. **d** Protein expression of Bax, Bcl-2 and Cyt-C in Bel-7402 were analyzed using Western blotting assay. **e** The expression of active caspase-3 was detected by IHC. **f**–**h** mRNA levels of Bax, Bcl-2 and Cyt-C in Huh-7 were determined using RT-qPCR. **i** Protein expressions of Bax, Bcl-2 and Cyt-C in Bel-7402 were determined using Western blotting assay. **j** The expression of active caspase-3 was detected by IHC. Data were shown as mean ± S.D. for three independent experiments. ^*^P < 0.05, ^**^P < 0.01 versus control

Furthermore, compared to control cells, as a typical pro-apoptotic gene, Bax was detected to be high-expressed in Bel-7402 cells in EEL groups. However, Bcl-2 and Cytochrome-C expression were obviously down-regulated in EEL groups in comparison to the control (Fig. 2a–d). Results from immunocytochemistry showed that Caspase-3 was limitedly expressed in normal Bel-7402 and Huh-7 cells, however, its expression increased in EEL groups (Fig. 2e). Meanwhile, the effect of EEL on Huh-7 cells was similar to that on Bel-7402 cells (Fig. 2f–j). It might be a limitation not using other quantitative measures of caspase 3 activity, but IHC assay also reflected the differences. In addition, compared with the control groups, Ki67, Cyclin D1 and p21 were found to be substantially reduced in HCC cell lines in EEL groups, particularly when EEL concentration was high (*P *< 0.05; Additional file 2: Figure S2).

### EEL suppressed migration and invasion of Bel-7402 and Huh-7 cell lines

**Fig. 3:**
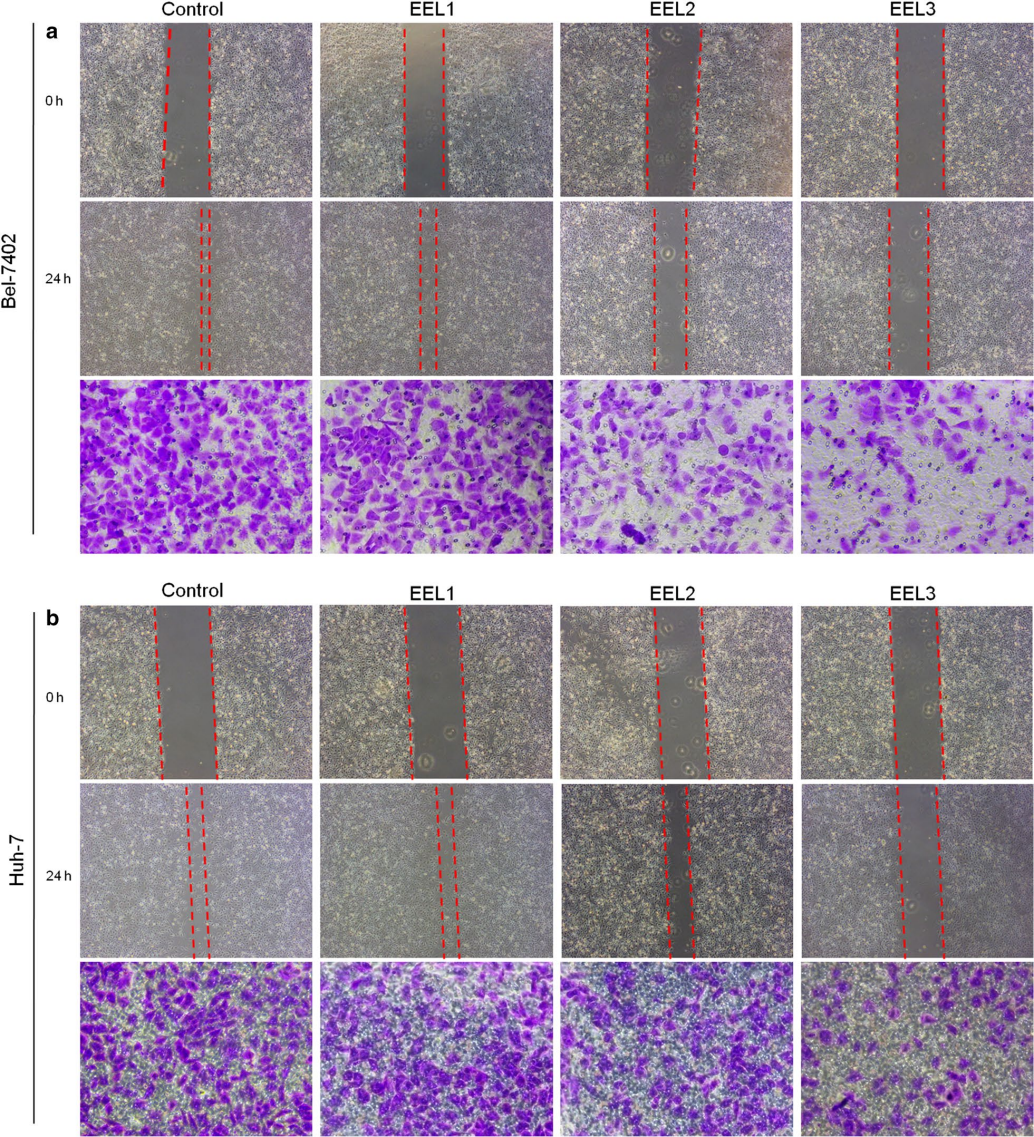
Effect of EEL treatment on cell migration and invasion. Bel-7402 and Huh-7 cells in control, EEL1, EEL2 and EEL3 groups were respectively treated with 0, 25, 50 and 75 mg/ml EEL for 24 h. **a** Cell migration and invasion of Bel-7402 was tested using cell wound scratch assay and transwell assay. **b** Cell migration and invasion of Huh-7 was tested using cell wound scratch assay and transwell assay. ^*^P < 0.05, ^**^P < 0.01 versus control

**Fig. 4:**
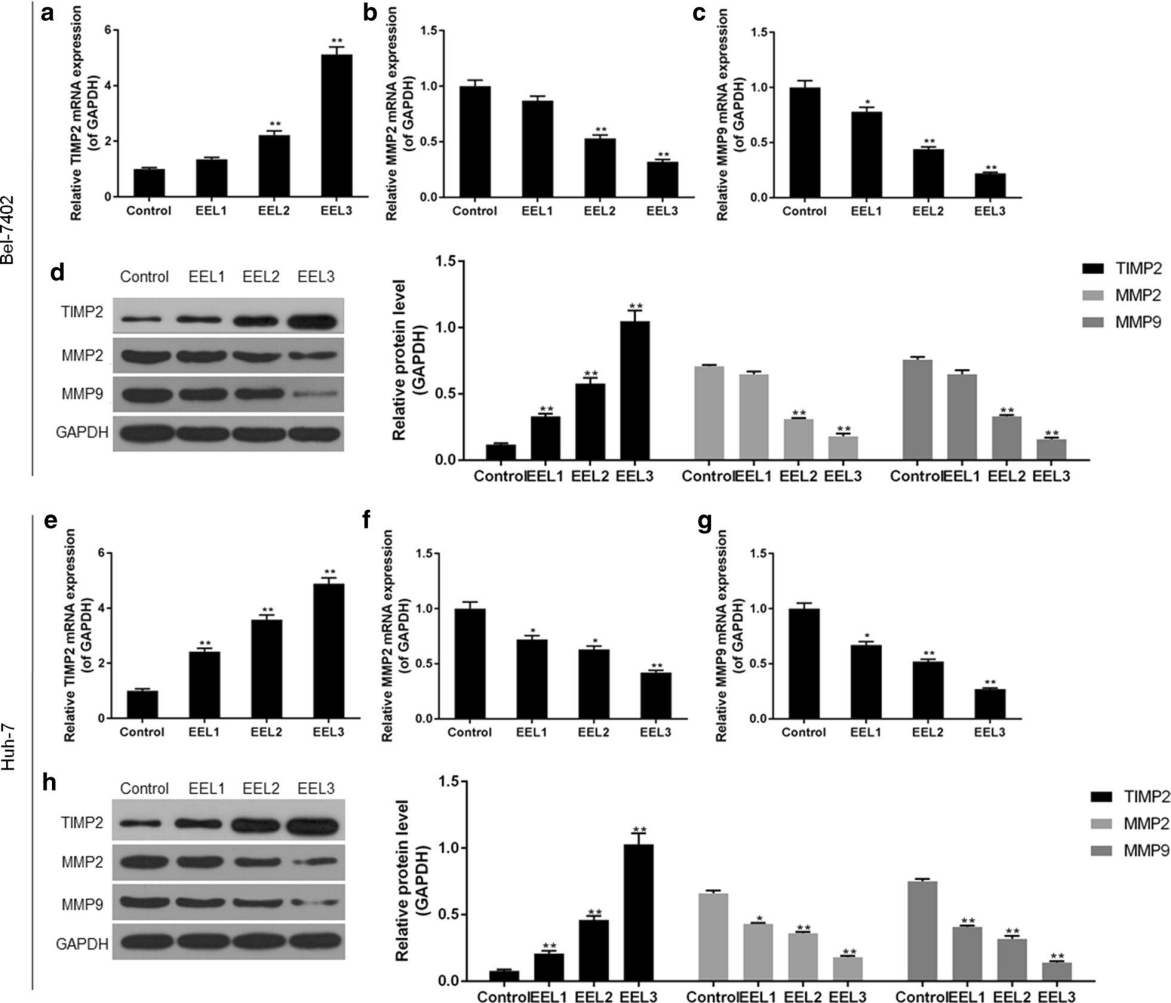
The effects of EEL treatment on the expression levels of TIMP2, MMP2 and MMP9. Bel-7402 and Huh-7 cells in control, EEL1, EEL2 and EEL3 groups were treated with respectively 0, 25, 50 and 75 mg/ml EEL for 24 h. **a**–**c** mRNA levels of TIM2, MMP2 and MMP9 in Bel-7402 were determined using RT-qPCR. **d** Protein expression of TIM2, MMP2 and MMP9 in Bel-7402 were analyzed using Western blotting assay. **e**–**g** mRNA levels of TIM2, MMP2 and MMP9 in Huh-7 were determined using RT-qPCR. **h** Protein expression of TIM2, MMP2 and MMP9 in Huh-7 were determined using Western blotting assay. Data were shown as mean ± S.D. for three independent experiments. ^*^P < 0.05, ^**^P < 0.01 versus control

Cell wound scratch assay and Transwell assay were performed to determine the effects of EEL treatment on cell migration and invasion of Bel-7402 and Huh-7 cell lines. As shown in Fig. 3, the results showed that EEL treatment could inhibit the migration and invasion of Bel-7402 and Huh-7 cells in a dose-dependent manner. We found that cell migration and invasion were obviously mitigated in EEL groups, compared with the control group. To further verify the results, the expression levels of TIMP2, MMP2 and MMP9 were determined, and we found that compared with control group, TIMP2 expression was up-regulated, whereas the expression levels of MMP2 and MMP9 were down-regulated in EEL groups (*P *< 0.05; Fig. 4).

### EEL inhibited the PI3K/Akt pathway

**Fig. 5:**
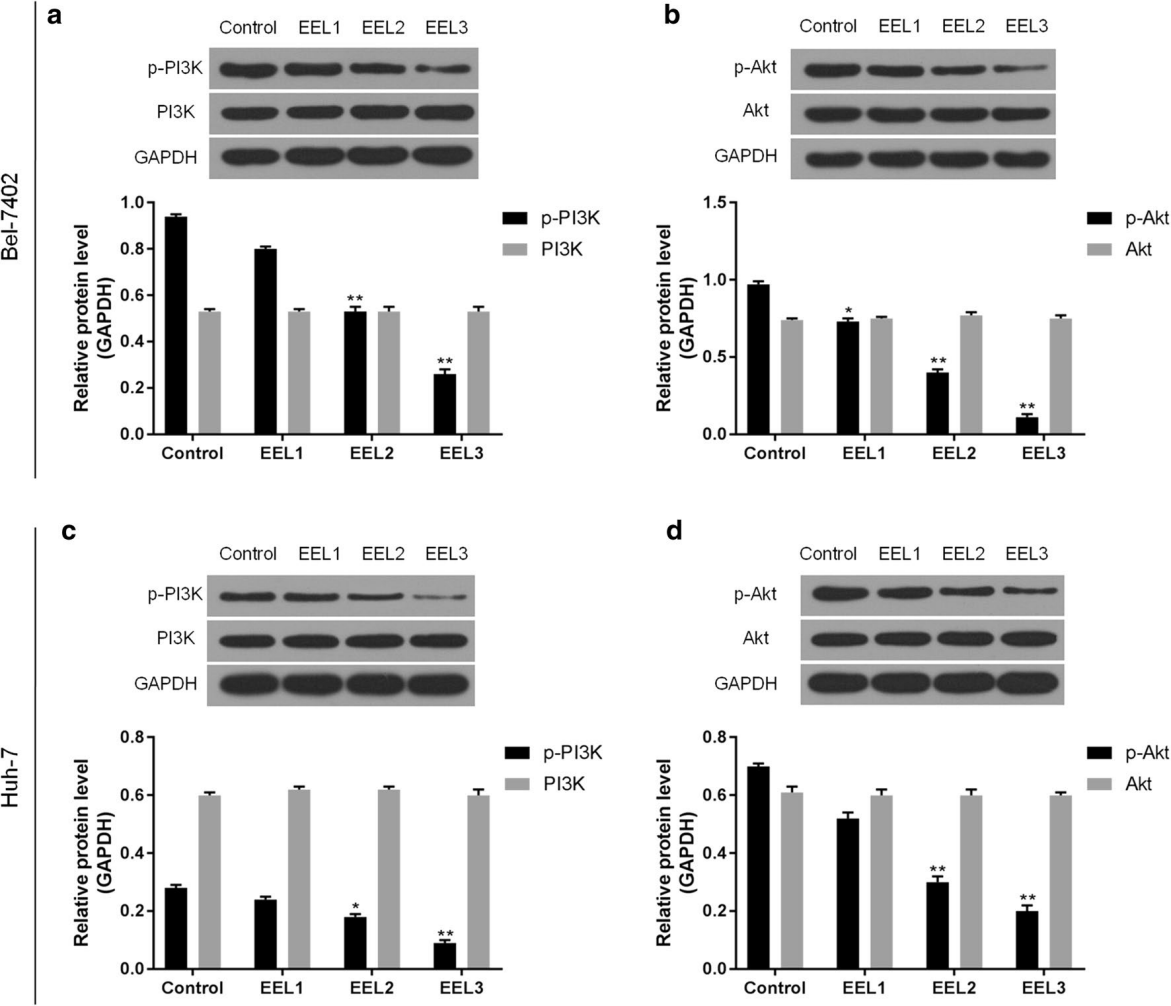
The effects of EEL treatment on the activity of PI3K/Akt pathway. Bel-7402 and Huh-7 cells in control, EEL1, EEL2 and EEL3 groups were respectively treated with 0, 25, 50 and 75 mg/ml EEL for 24 h. **a**, **b** Phosphorylation levels of PI3K and Akt in Bel-7402 were detected using Western blotting assay. **c**, **d** Phosphorylation levels of PI3K and Akt in Huh-7 were detected using Western blotting assay. Data were shown as mean ± S.D. for three independent experiments. ^*^P < 0.05, ^**^P < 0.01 versus control

**Fig. 6:**
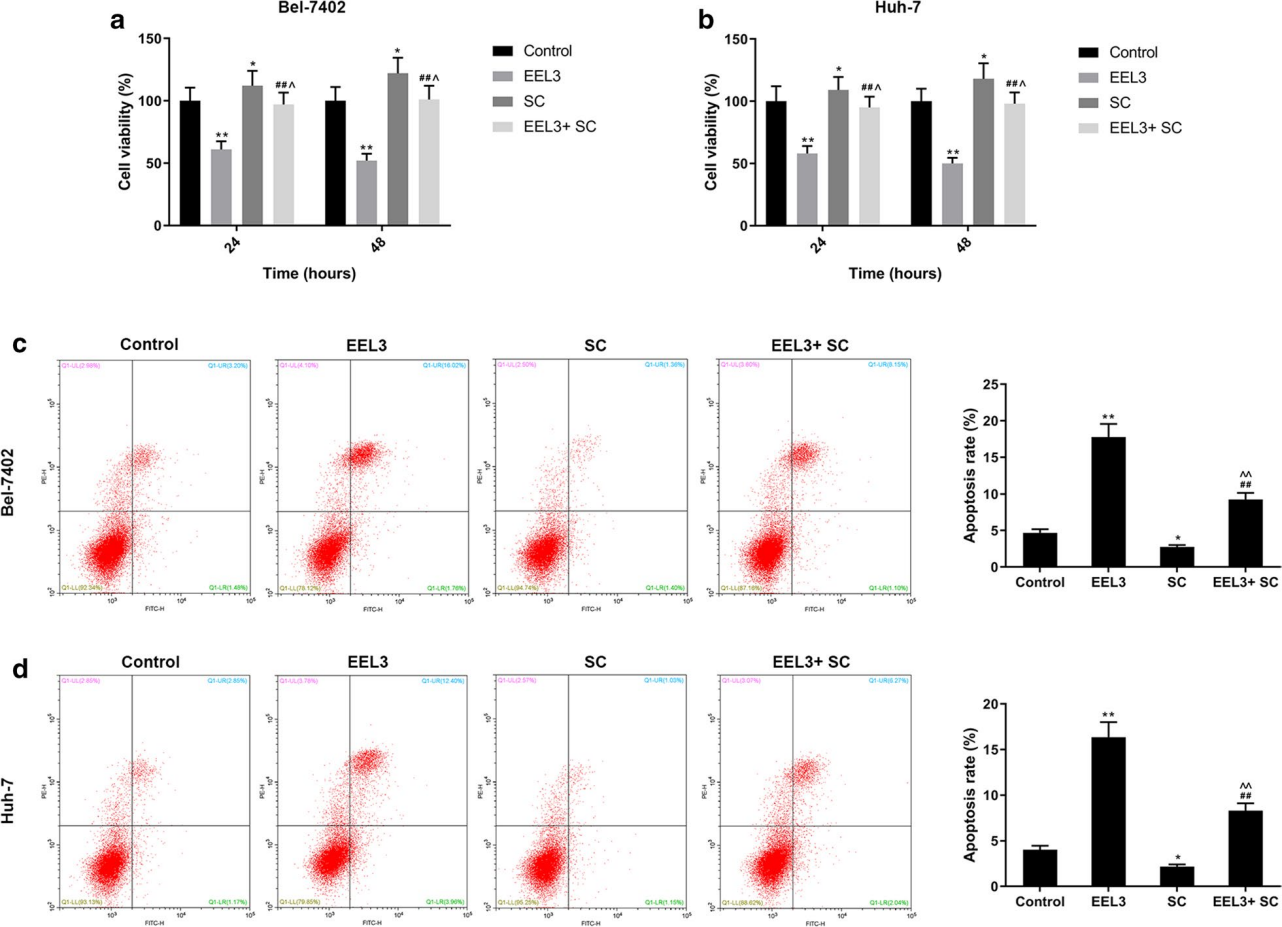
The effects of Akt activator on EEL treated HCC cells. **a**, **b** Cell viability in each group was determined using CCK-8 assay. **c**, **d** Cell apoptosis was detected using FCM. ^*^P < 0.05, ^**^P < 0.01 versus control. ^##^*P *< 0.01 versus EEL3. ^^^*P *< 0.05, ^^^^*P *< 0.01 versus SC

Western blotting assay showed that the protein levels of p-PI3K and p-Akt in EEL treatment groups were markedly down-regulated in HCC cell lines in comparison to the control group (Fig. 5). Additionally, the phosphorylation levels of PI3K and Akt were down-regulated noticeably as the EEL concentration increased. This suggested that the inhibition of PI3K/Akt pathway was possibly in association with the biological activity of EEL in HCC cells. After applying Akt activator SC79, the cell viability was improved in EEL3 + SC group, compared with EEL3 group, while cell apoptosis was decreased in EEL3 + SC group, compared with EEL3 group (Fig. 6).

### EEL suppressed DNA methylation of PTEN in Bel-7402 and Huh-7 cell lines

**Fig. 7:**
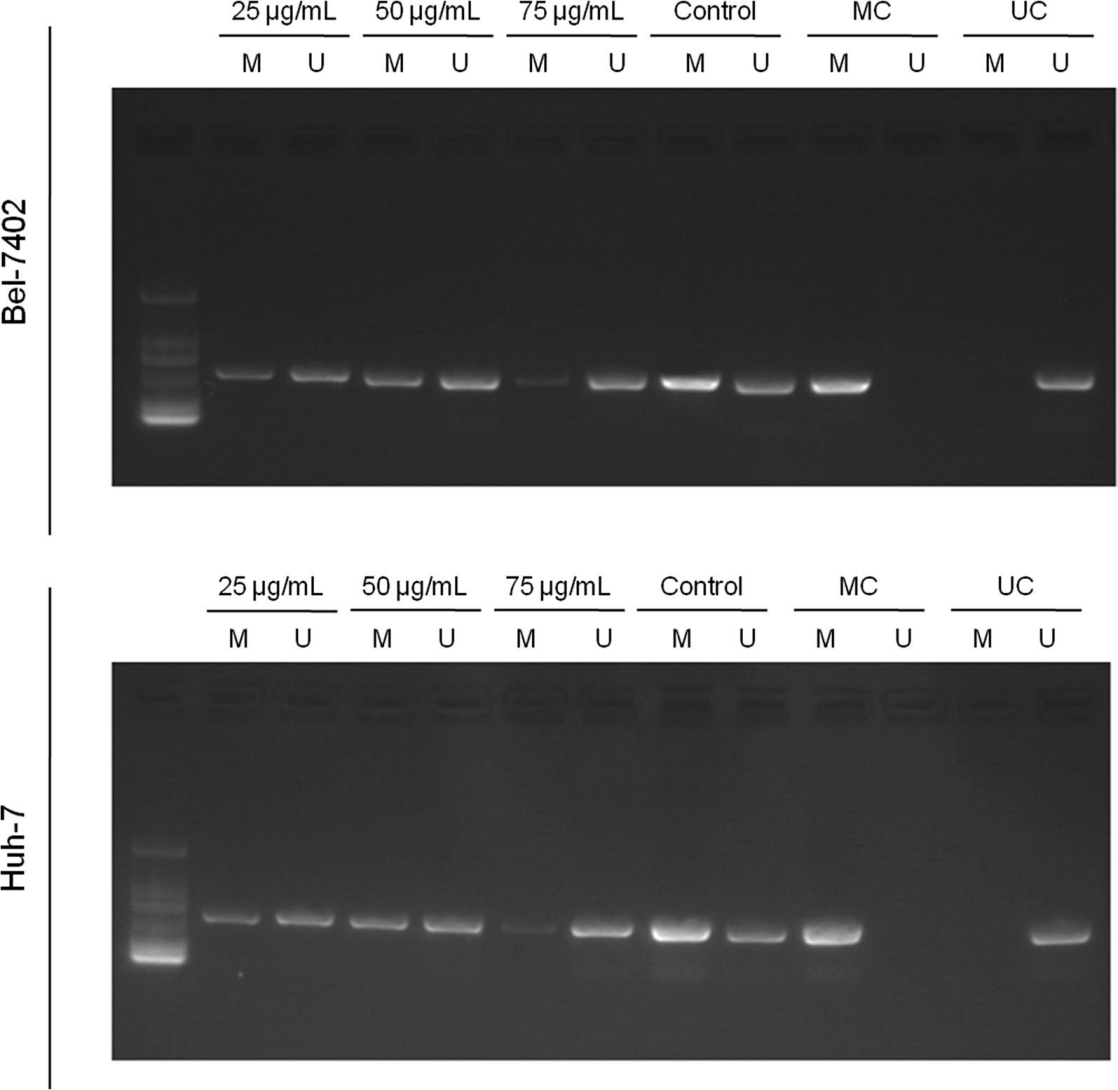
The effect of EEL treatment on DNA methylation of PTEN. Bel-7402 and Huh-7 cells in control, EEL1, EEL2 and EEL3 groups were treated with respectively 0, 25, 50 and 75 mg/ml EEL for 24 h. DNA methylation levels of PTEN in Bel-7402 and Huh-7 cell lines were determined using MSP. The abbreviations were methylated (M), unmethylated (U), methylated control (MC) or unmethylated control (UC)

MSP assay was performed to investigate the possible mechanism of EEL in HCC, and the results showed that methylated (M) and unmethylated (U) *PTEN* gene were both expressed in these two cell lines in the control groups, by contrast, the methylated levels were down-regulated in EEL groups. In the group with high concentration of EEL, we found that methylated PTEN was limitedly detected (Fig. 7). The MSP results showed that DNA methylation of PTEN was associated with EEL treatment.

### EEL inhibited the tumor growth of HCC in vivo

**Fig. 8:**
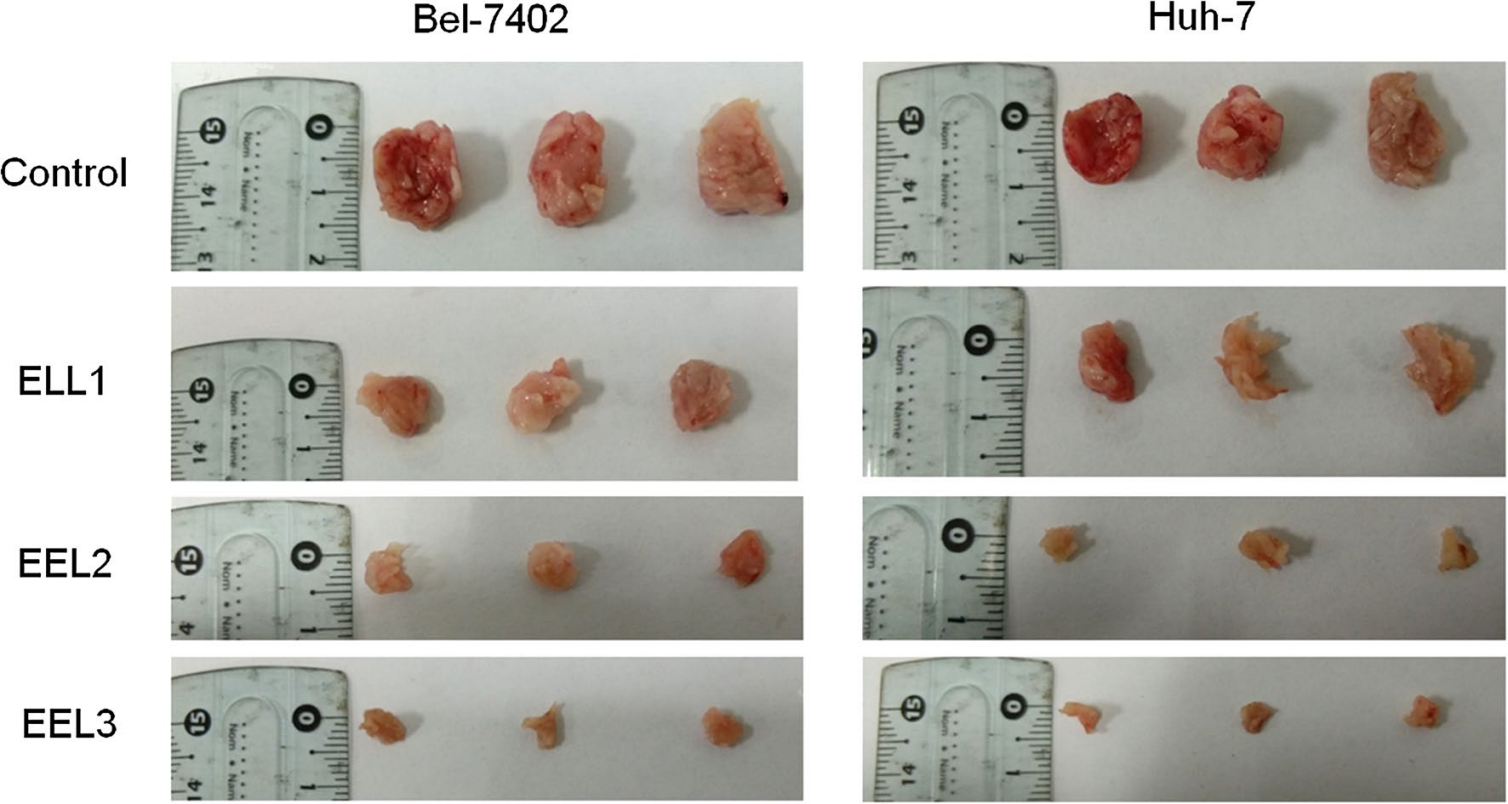
The effect of EEL treatment on tumor growth of HCC in vivo. EEL reduced the tumor diameter of HCC. The tumor-bearing mice were treated with 1.5 g/kg, 3 g/kg and 5 g/kg EEL. After 4 weeks, the tumor diameter was measured

We detected the tumor growth in vivo to further confirm the effect of EEL on HCC, and the results in Fig. 8 revealed that the tumor diameter was shortened in a dose-dependent manner by the treatment of EEL.

## Discussion

IEEL is a typical Chinese medicine that has been widely prescribed to treat renal diseases and was recently reported to have a pro-apoptotic ability in human HCC cell line [14–16]. *Ligustrum lucidum Ait*. fruit extract induced apoptosis and cell senescence in human hepatocellular carcinoma cells through upregulation of p21 [16]. Specnuezhenide, an effective constituent of *Ligustrum lucidum Ait*., inhibited hypoxia-Induced retinal angiogenesis through suppression of the HIF-1alpha/VEGF signaling pathway [15]. On such basis, our study further probed into the role of EEL on HCC and verified the possible mechanism of EEL in HCC.

Our study proved that EEL delivered cytotoxicity to both Bel-7402 and Huh-7 cell lines in a concentration-dependent manner. We also discovered that EEL could evidently induce cell apoptosis of Bel-7402 and Huh-7 cells by regulating the expression of Cytochrome-C and the levels of Bcl-2 and Bax. Data from immunocytochemistry assay showed that the activity of caspase-3 was increased by EEL. G1 cell cycle arrest of Bel-7402 and Huh-7 was also observed in EEL groups, compared with the control group. Moreover, the expression levels of Ki67, Cyclin D1 and p21 were substantially down-regulated by the treatment of EEL. Furthermore, suppression of migration and invasion rates was inhibited by EEL. We also observed the up-regulation of TIM2 expression and down-regulation of MMP2 and MMP9 levels in EEL groups, which further proved the inhibiting ability of EEL on cancer metastasis. It might be a limitation not using the restorative expression to address which of those factors are crucial for EEL, and we might continue studying it in future study. Researches has demonstrated that PI3K/Akt pathway played a key role in cancer cells [17, 18]. The signal pathway can be activated by extracellular stimulating factor through G protein-coupled receptors [19, 20]. Upon the phosphorylation of PI3K, PIP3, the second messenger will activate the phosphorylation of Akt, and phosphorylated Akt then regulates the biological activities of downstream targets [21, 22]. To investigate the mechanism under the EEL treatment, we speculated that PI3K/PTEN/Akt pathway might be involved in the effect of EEL, and our data showed that the expressions of p-PI3K and p-Akt in HCC cell lines were reduced by the treatment of EEL. After applying Akt activator SC79 on HCC cells treated with EEL, cell viability was improved and cell apoptosis was decreased.

As one of the genome epigenetic modifications, DNA methylation is a key contributor to stable genetic expression and exists in higher organisms [23]. In the process of DNA methylation, a methyl in the donor S-adenosyl-l-methionine (SAM) will be transferred into specific basic groups with the help of DNA methyltransferases (DNMTs) [24, 25]. In eukaryotic cells from higher organisms, the object of methylated modification is cytosine in cytosine-phosphate-guanine (CpG) sequence, and noticeably, 60% of human gene promoters are closely related to CpG islands [26–28]. It has been reported that DNA methylation could lead to transcriptional silencing of PTEN [29]. PTEN could not only dephosphorylate PI(3,4,5)P3 to PI(4,5)P2 and PI(3,4)P2 to PI(4)P and cause the down-regulation of PI3K/Akt pathway [30–35], but also suppress upstream proteins of PI3K [32]. By MSP method, we found that EEL could down-regulate the DNA methylation level of PTEN, and that the expression of unmethylated PTEN increased, however, the methylated levels were down-regulated in EFL groups, and noticeably, we found that in the group with a high concentration of EEL, methylated PTEN was limitedly detected and only unmethylated PTEN was clearly expressed, which demonstrated the demethylation ability of EEL in Bel-7402 and HUH-7 cell lines. Thus, out data indicated that the inactivation of PI3K/Akt pathway may be related to de-methylation of PTEN. A previous study has reported that p-Akt level was evidently up-regulated in prostate cancer under PTEN inactivation [34]. We also demonstrated that the tumor growth of HCC was inhibited by EEL in vivo. Taken together, our data proved that EFL exerted anti-tumor effect in HCC and the inhibition of PI3K/Akt may be related to the effect of EFL, and that the demethylation of PTEN caused by EFL may contribute to the suppression of PI3K/Akt pathway.

We also detected the tumor growth in vivo to further confirm the effect of EEL on HCC, and the study revealed that the tumor diameter was shortened in a dose-dependent manner by the treatment of EEL. It might be a limitation not establishing animal tumor model to see if EEL suppresses tumor growth, and we might continue studying it in future study.

## Conclusion

In conclusion, our study proved that EEL suppressed the cell proliferation of HCC cells. EEL induced apoptosis and promoted cell cycle arrest by regulating the expressions of factors (Bax, Bcl-2, cytC, caspase-3, ki67, cyclinD1 and P21) related to apoptosis and cell cycle arrest. EEL reduced the invasion and migration of HCC cells and modulated the expressions of TIMP and MMP2/9 and suppressed the activation of PI3K/Akt pathway. Significantly, EEL enhanced the de-methylation of PTEN, which possibly contribute to the inhibition of PI3K/Akt pathway. Our data suggested that EEL could be used as a potential anti-cancer agent of HCC. This study provides a potential therapeutic strategy for the treatment of HCC.

## Supplementary information

**Additional file 1: Figure S1.** Effect of EEL on viability of Bel-7402 nd Huh-7 cells. After being treated with different concentrations of EEL (5, 10, 25, 50, 75, and 100 mg/ml), cell viability in each group was determined using CCK-8 assay at 12, 24 and 48 h. Data were shown as mean ± S.D for three independent experiments. ^*^P < 0.05, ^**^P < 0.01 versus control.

**Additional file 2: Figure S2.** Effect of EEL treatment on the expression levels of cell cycle-related genes. Bel-7402 and Huh-7 cells in control, EEL1, EEL2 and EEL3 groups were treated respectively with 0, 25, 50 and 75 mg/ml EEL for 24 h. Data in A–D were generated with Bel-7402 cells, while data in E–H were produced with Huh-7 cells. (A–C) mRNA levels of Ki67, Cyclin D1 and p21 in Bel-7402 were determined using RT-qPCR. (D) Protein expressions of Ki67, Cyclin D1 and p21 in Bel-7402 were analyzed using Western blotting assay. (E–G) mRNA levels of Ki67, Cyclin D1 and p21 in Huh-7 were determined using RT-qPCR. (H) Protein expressions of Ki67, Cyclin D1 and p21 in Huh-7 were determined using Western blotting assay. Data were shown as mean ± S.D. for three independent experiments. ^*^P < 0.05, ^**^P < 0.01 versus control.

## Data Availability

The analyzed data sets generated during the study are available from the corresponding author on reasonable request.

## Abbreviations

EEL: ethanol extract of *Ligustrum lucidum Ait*. leaves
CCK-8: cell counting kit-8
FCM: flow cytometry
MSP: methylation-specific PCR
qRT-PCR: quantitative real-time PCR
Bax: Bcl-2 assaciated X
Bcl-2: B cell lymphoma 2
TIMP2: tissue inhibitor of metalloproteinases 2
MMP2: matrix metalloproteinase2
HCC: hepatocellular carcinoma
GAPDH: glyceraldehyde-3-phosphate dehydrogenase
SDS-PAGE: sodium dodecyl sulfatepolyacrylamide gel electrophoresis
ANOVA: analysis of variance

## References

[1] Ferlay J, Soerjomataram I, Dikshit R, Eser S, Mathers C, Rebelo M, Parkin DM, Forman D, Bray F (2015). Cancer incidence and mortality worldwide: sources, methods and major patterns in GLOBOCAN 2012. Int J Cancer.

[2] Fujiyama S, Shibata J, Maeda S, Tanaka M, Noumaru S, Sato K, Tomita K (2003). Phase I clinical study of a novel lipophilic platinum complex (SM-11355) in patients with hepatocellular carcinoma refractory to cisplatin/lipiodol. Br J Cancer.

[3] Okusaka T, Okada S, Nakanishi T, Fujiyama S, Kubo Y (2004). Phase II trial of intra-arterial chemotherapy using a novel lipophilic platinum derivative (SM-11355) in patients with hepatocellular carcinoma. Invest New Drugs.

[4] Tian H, Ge C, Zhao F, Zhu M, Zhang L, Huo Q, Li H, Chen T, Xie H, Cui Y (2017). Downregulation of AZGP1 by Ikaros and histone deacetylase promotes tumor progression through the PTEN/Akt and CD44s pathways in hepatocellular carcinoma. Carcinogenesis.

[5] Yu M, Mu Y, Qi Y, Qin S, Qiu Y, Cui R, Zhong M (2016). Odontogenic ameloblast-associated protein (ODAM) inhibits human colorectal cancer growth by promoting PTEN elevation and inactivating PI3K/AKT signaling. Biomed Pharmacother.

[6] Xiao J, Yu W, Hu K, Li M, Chen J, Li Z (2017). miR-92a promotes tumor growth of osteosarcoma by targeting PTEN/AKT signaling pathway. Oncol Rep.

[7] Zhang X, Chen Y, Zhao P, Zang L, Zhang Z, Wang X (2017). MicroRNA-19a functions as an oncogene by regulating PTEN/AKT/pAKT pathway in myeloma. Leukemia Lymphoma.

[8] Li C, Liang G, Yang S, Sui J, Wu W, Xu S, Ye Y, Shen B, Zhang X, Zhang Y (2019). LncRNA-LOC101928316 contributes to gastric cancer progression through regulating PI3K-Akt-mTOR signaling pathway. Cancer Med.

[9] Tang S, Li Y, Bao Y, Dai Z, Niu T, Wang K, He H, Song D (2019). Novel cytisine derivatives exert anti-liver fibrosis effect via PI3K/Akt/Smad pathway. Bioorg Chem.

[10] Getahun A, Wemlinger SM, Rudra P, Santiago ML, van Dyk LF, Cambier JC (2017). Impaired B cell function during viral infections due to PTEN-mediated inhibition of the PI3 K pathway. J Exp Med.

[11] Sun L, Yu F, Yi F, Xu L, Jiang B, Le L, Xiao P (2019). Acteoside from *Ligustrum robustum* (Roxb.) blume ameliorates lipid metabolism and synthesis in a HepG2 cell model of lipid accumulation. Front Pharmacol.

[12] Qiu ZC, Zhao XX, Wu QC, Fu JW, Dai Y, Wong MS, Yao XS (2018). New secoiridoids from the fruits of *Ligustrum lucidum*. J Asian Nat Prod Res.

[13] Wang C, Gao H, Cai E, Zhang L, Zheng X, Zhang S, Sun N, Zhao Y (2019). Protective effects of *Acanthopanax senticosus*–*Ligustrum lucidum* combination on bone marrow suppression induced by chemotherapy in mice. Biomed Pharmacother.

[14] Zhang L, Ravipati AS, Koyyalamudi SR, Jeong SC, Reddy N, Bartlett J, Smith PT, de la Cruz M, Monteiro MC, Melguizo A (2013). Anti-fungal and anti-bacterial activities of ethanol extracts of selected traditional Chinese medicinal herbs. Asian Pac J Trop Med.

[15] Wu J, Ke X, Fu W, Gao X, Zhang H, Wang W, Ma N, Zhao M, Hao X, Zhang Z (2016). Inhibition of hypoxia-induced retinal angiogenesis by specnuezhenide, an effective constituent of *Ligustrum lucidum Ait.*, through suppression of the HIF-1alpha/VEGF signaling pathway. Molecules.

[16] Hu B, Du Q, Deng S, An HM, Pan CF, Shen KP, Xu L, Wei MM, Wang SS (2014). *Ligustrum lucidum Ait.* fruit extract induces apoptosis and cell senescence in human hepatocellular carcinoma cells through upregulation of p21. Oncol Rep.

[17] Barone I, Cui Y, Herynk MH, Coronarodriguez A, Giordano C, Selever J, Beyer A, Andò S, Fuqua SA (2009). Expression of the K303R estrogen receptor α breast cancer mutation induces resistance to an aromatase inhibitor via addiction to the PI3K/Akt kinase pathway. Can Res.

[18] Vara JÁ, Casado E, de Castro J, Cejas P, Belda-Iniesta C, González-Barón M (2004). PI3K/Akt signalling pathway and cancer. Cancer Treat Rev.

[19] Guillermet-Guibert J, Bjorklof K, Salpekar A, Gonella C, Ramadani F, Bilancio A, Meek S, Smith AJ, Okkenhaug K, Vanhaesebroeck B (2008). The p110beta isoform of phosphoinositide 3-kinase signals downstream of G protein-coupled receptors and is functionally redundant with p110gamma. Proc Natl Acad Sci USA.

[20] Schmid MC, Avraamides CJ, Dippold HC, Franco I, Foubert P, Ellies LG, Acevedo LM, Manglicmot JR, Song X, Wrasidlo W (2011). Receptor tyrosine kinases and TLR/IL1Rs unexpectedly activate myeloid cell PI3kgamma, a single convergent point promoting tumor inflammation and progression. Cancer Cell.

[21] Padala RR, Karnawat R, Viswanathan SB, Thakkar AV, Das AB (2017). Cancerous perturbations within the ERK, PI3K/Akt, and Wnt/beta-catenin signaling network constitutively activate inter-pathway positive feedback loops. Mol BioSyst.

[22] Xuan W, Feng X, Qian C, Peng L, Shi Y, Xu L, Wang F, Tan W (2017). Osteoclast differentiation gene expression profiling reveals chemokine CCL4 mediates RANKL-induced osteoclast migration and invasion via PI3K pathway. Cell Biochem Funct.

[23] Jaenisch R, Bird A (2003). Epigenetic regulation of gene expression: how the genome integrates intrinsic and environmental signals. Nat Genet.

[24] Huang Y, Tan H, Cao Q, Yuan G, Su G, Yang P (2019). Different methylation of CpG-SNPs in Behcet’s disease. Biomed Res Int.

[25] Traube FR, Carell T (2017). The chemistries and consequences of DNA and RNA methylation and demethylation. RNA Biol.

[26] Bird A (2002). DNA methylation patterns and epigenetic memory. Genes Dev.

[27] Goll MG, Bestor TH (2005). Eukaryotic cytosine methyltransferases. Annu Rev Biochem.

[28] Straussman R, Nejman D, Roberts D, Steinfeld I, Blum B, Benvenisty N, Simon I, Yakhini Z, Cedar H (2009). Developmental programming of CpG island methylation profiles in the human genome. Nat Struct Mol Biol.

[29] Lubecka-Pietruszewska K, Kaufman-Szymczyk A, Stefanska B, Fabianowska-Majewska K (2013). Folic acid enforces DNA methylation-mediated transcriptional silencing of PTEN, APC and RARbeta2 tumour suppressor genes in breast cancer. Biochem Biophys Res Commun.

[30] Stocker H, Andjelkovic M, Oldham S, Laffargue M, Wymann MP, Hemmings BA, Hafen E (2002). Living with lethal PIP3 levels: viability of flies lacking PTEN restored by a PH domain mutation in Akt/PKB. Science.

[31] Wu X, Hepner K, Castelino-Prabhu S, Do D, Kaye MB, Yuan XJ, Wood J, Ross C, Sawyers CL, Whang YE (2000). Evidence for regulation of the PTEN tumor suppressor by a membrane-localized multi-PDZ domain containing scaffold protein MAGI-2. Proc Natl Acad Sci USA.

[32] Schneider E, Keppler R, Prawitt D, Steinwender C, Roos FC, Thuroff JW, Lausch E, Brenner W (2011). Migration of renal tumor cells depends on dephosphorylation of Shc by PTEN. Int J Oncol.

[33] Goo CK, Lim HY, Ho QS, Too HP, Clement MV, Wong KP (2012). PTEN/Akt signaling controls mitochondrial respiratory capacity through 4E-BP1. PLoS ONE.

[34] Hua S, Yao M, Vignarajan S, Witting P, Hejazi L, Gong Z, Teng Y, Niknami M, Assinder S, Richardson D (2013). Cytosolic phospholipase A2alpha sustains pAKT, pERK and AR levels in PTEN-null/mutated prostate cancer cells. Biochem Biophys Acta.

[35] Bai ZG, Ye YJ, Shen DH, Lu YY, Zhang ZT, Wang S (2013). PTEN expression and suppression of proliferation are associated with Cdx2 overexpression in gastric cancer cells. Int J Oncol.

