# Supplementary figures and images for: Ethanol extract of Ligustrum lucidum Ait. leaves suppressed hepatocellular carcinoma in vitro and in vivo
Source: Cancer Cell Int. 2019 Sep 26;19:246. doi: 10.1186/s12935-019-0960-5 (PMC6761729; doi:10.1186/s12935-019-0960-5)

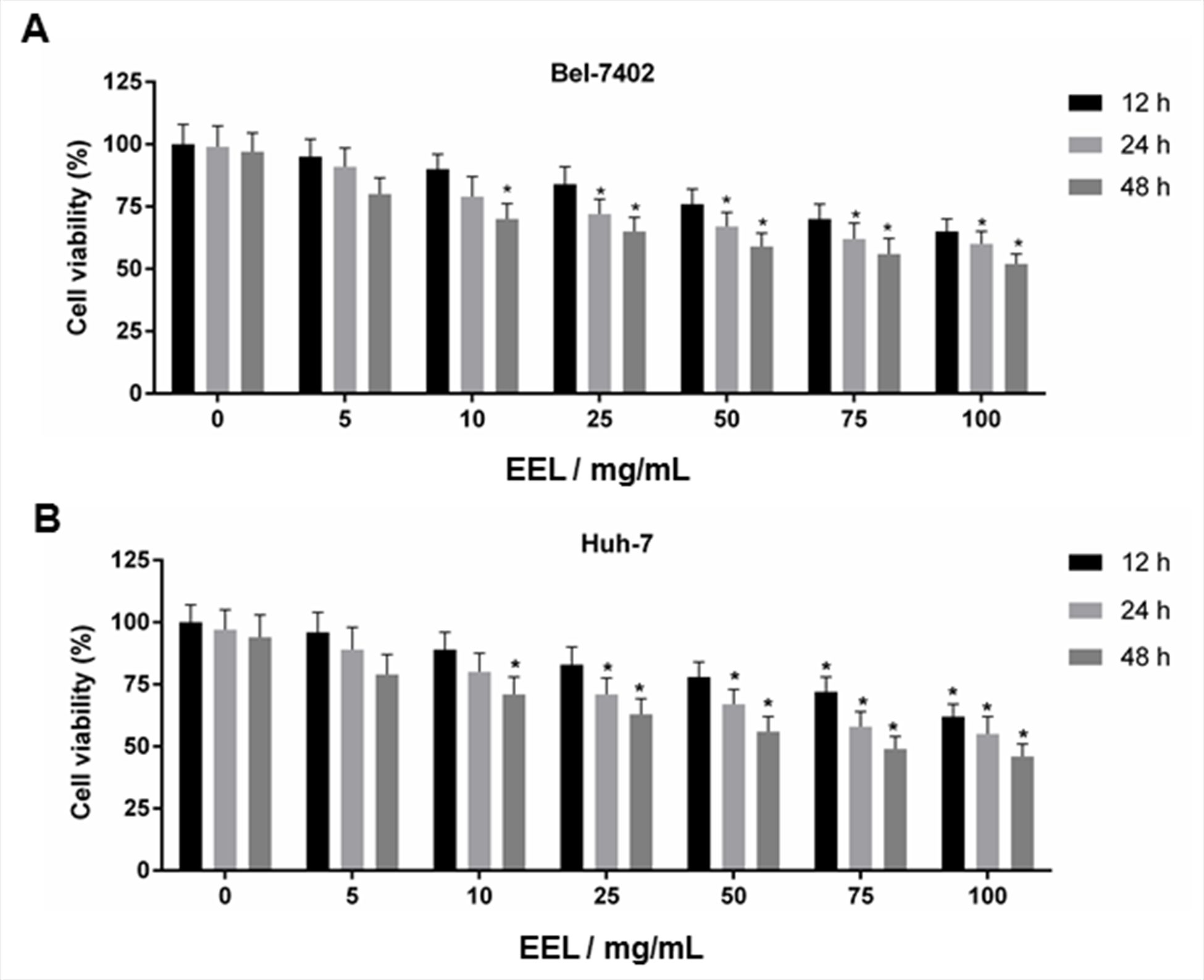

Supplement: Supplementary file 1 — Additional file 1: Figure S1. Effect of EEL on viability of Bel-7402 nd Huh-7 cells. After being treated with different concentrations of EEL (5, 10, 25, 50, 75, and 100 mg/ml), cell viability in each group was determined using CCK-8 assay at 12, 24 and 48 h. Data were shown as mean ± S.D for three independent experiments. *P < 0.05, **P < 0.01 versus control. [file 12935_2019_960_MOESM1_ESM.tif]

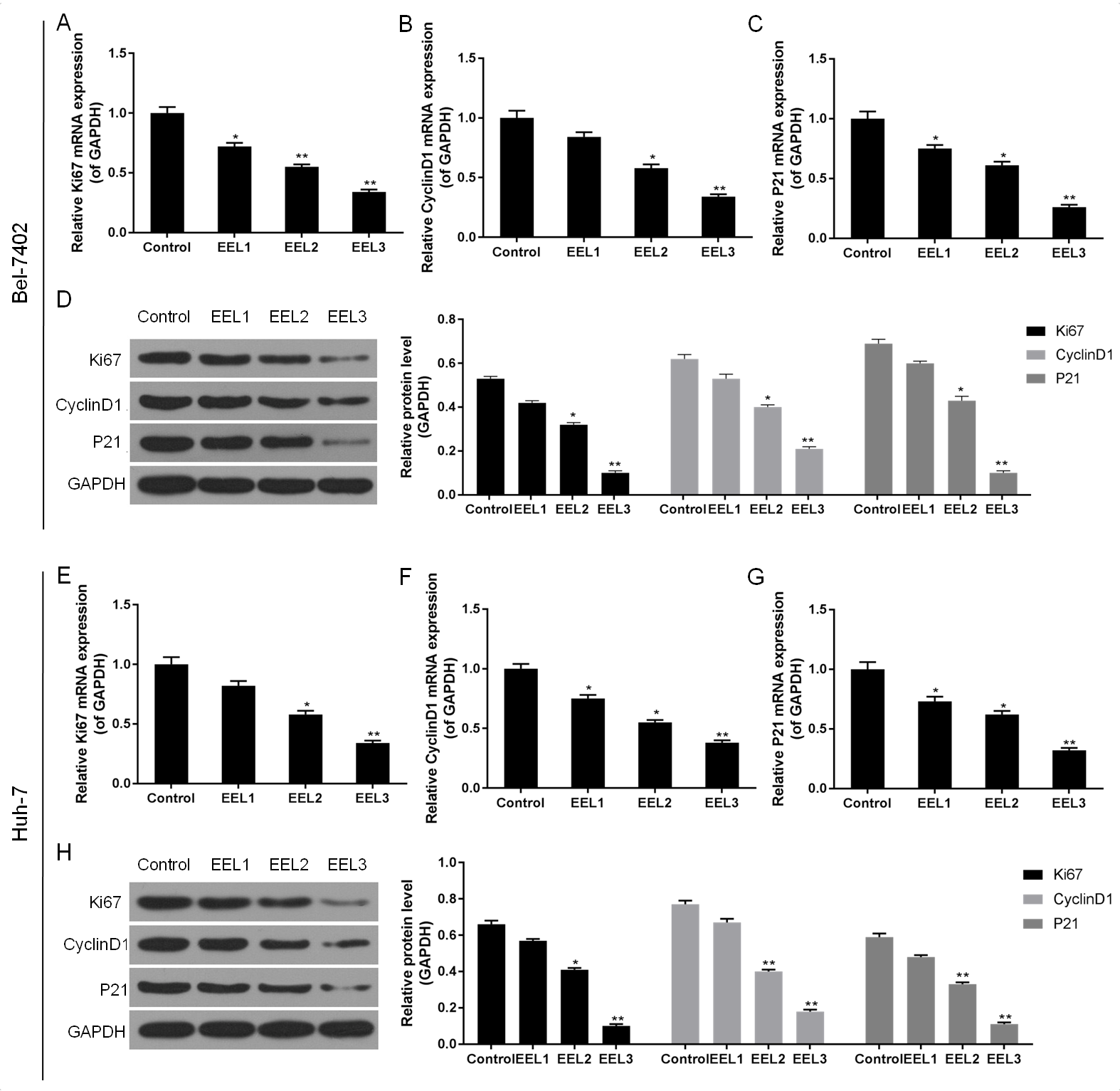

Supplement: Supplementary file 2 — Additional file 2: Figure S2. Effect of EEL treatment on the expression levels of cell cycle-related genes. Bel-7402 and Huh-7 cells in control, EEL1, EEL2 and EEL3 groups were treated respectively with 0, 25, 50 and 75 mg/ml EEL for 24 h. Data in A–D were generated with Bel-7402 cells, while data in E–H were produced with Huh-7 cells. (A–C) mRNA levels of Ki67, Cyclin D1 and p21 in Bel-7402 were determined using RT-qPCR. (D) Protein expressions of Ki67, Cyclin D1 and p21 in Bel-7402 were analyzed using Western blotting assay. (E–G) mRNA levels of Ki67, Cyclin D1 and p21 in Huh-7 were determined using RT-qPCR. (H) Protein expressions of Ki67, Cyclin D1 and p21 in Huh-7 were determined using Western blotting assay. Data were shown as mean ± S.D. for three independent experiments. *P < 0.05, **P < 0.01 versus control. [file 12935_2019_960_MOESM2_ESM.tif]
